# Association of superficial macular vessel density with visual field progression in open-angle glaucoma with central visual field damage

**DOI:** 10.1038/s41598-023-34000-6

**Published:** 2023-05-03

**Authors:** Jooyoung Yoon, Anna Lee, Woo Keun Song, Ko Eun Kim, Michael S. Kook

**Affiliations:** Department of Ophthalmology, Asan Medical Center, University of Ulsan College of Medicine, 88 Olympic-Ro 43-Gil, Songpa-Gu, Seoul, South Korea

**Keywords:** Glaucoma, Prognostic markers

## Abstract

Identifying the clinical relevance of superficial versus deep layer macular vessel density (mVD) in glaucoma is important for monitoring glaucoma patients. Our current retrospective longitudinal study investigated the association of superficial and deep layer mVD parameters with glaucomatous visual field (VF) progression in mild to moderate open-angle glaucoma (OAG) eyes with central visual field (CVF) damage. Serial optical coherence tomography (OCT) angiography-derived mVD measurements were obtained in 182 mild to moderate OAG eyes (mean deviation ≥ -10 decibels). Forty-eight eyes (26.4%) showed VF progression during a mean follow-up of 3.5 years. The parafoveal and perifoveal mVDs of both superficial and deep layers showed significantly faster reduction rates in the VF progressors than in the non-progressors according to linear mixed effects models (*P* < 0.05). Cox and linear regression analyses showed that greater reduction rates of both the superficial layer parafoveal and perifoveal mVDs, but not their deep layer counterparts, were significant predictors of VF progression and faster VF loss (*P* < 0.05). In conclusion, faster rates of change in superficial but not deep layer mVD parameters are significantly associated with subsequent VF progression and faster VF deterioration in mild to moderate OAG eyes with CVF damage.

## Introduction

Central visual field (CVF) defects can develop in early glaucoma patients^1,2^, and have considerable negative impacts on daily activities, such as reading, household chores, and outdoor activities^3^. Moreover, eyes with initial CVF defects show more rapid global visual field (VF) progression^4^. Hence, the early detection and prevention of CVF progression is crucial even in early-stage glaucoma patients, and numerous attempts have thus been made to elucidate the factors associated with CVF damage. For example, risk factors associated with CVF scotoma may include optic disc haemorrhage (ODH), systemic hypotension, Raynaud’s phenomenon, and migraine, suggesting that vascular insufficiency may play an important role in the development of CVF damage^2^.

The recent development of optical coherence tomography angiography (OCT-A) technology has enabled clinicians to conduct in vivo visualizations of the microvasculatures of the optic nerve head (ONH), circumpapillary retina, and macular area, and to correlate retinal micro-perfusion in different layers and locations of a retina with glaucomatous damage in an objective and quantitative manner^5–11^. The current OCT-A devices provide two-layer (i.e., superficial [SVP] and deep vascular plexus [DVP]) segmentation slabs in the macula, based on the anatomical array of the retina. Since the retinal ganglion cells (RGCs) and their axons are located in the inner retina, several studies have reported that glaucoma preferentially affects the SVP more than the DVP^5,12^, and that the macular vessel density (mVD) in the SVP shows better glaucoma diagnostic capabilities than that in the DVP^5,9^.

While few studies to date using OCT-A have demonstrated that the mVD in the deep layer of glaucoma patients has a stronger correlation with CVF damage compared to that in the superficial layer^6,7^, knowledge of the clinical relevance of the different macular layers on monitoring disease progression has remained limited in glaucoma patients with CVF damage. Of interest in this regard, Kamalipour et al^8^. recently demonstrated that the lower superficial mVD, but not deep mVD, was associated with faster VF progression prior to OCT-A measurements in glaucomatous eyes with and without initial CVF defects. Nonetheless, there is currently no report that has revealed the relationship between longitudinal changes in the mVD at different layers and concurrent VF progression in glaucomatous eyes. Hence, the aim of our present study was to evaluate the correlations between mVD reduction at different layers (superficial vs. deep layer) and concomitant VF progression in mild to moderate stage OAG eyes with CVF defects.

## Methods

### Patients

The protocols of this study were reviewed and approved by the institutional review board (IRB) of Asan Medical Center which waived the requirement for written informed consent from the study subjects due to the retrospective nature of the analyses (IRB approval number: 2018–0008). This retrospective study adhered to the tenets of the Declaration of Helsinki. Medical records were obtained and reviewed consecutively from patients who visited the glaucoma clinic of the Asan Medical Center from April 2018 to November 2021.

All study patients underwent comprehensive ophthalmologic examinations at their initial visit, including measurement of best-corrected visual acuity (BCVA), intraocular pressure (IOP) by Goldmann applanation tonometry, central corneal thickness (CCT) with ultrasonic pachymetry (Tomey SP-3000, Nagoya, Japan), axial length using an IOL master (Carl Zeiss Meditec, Inc., Dublin, CA), slit-lamp biomicroscopy, gonioscopy, dilated colour fundus photography (Canon, Tokyo, Japan), optic disc stereoscopic photography, red-free retinal nerve fibre layer (RNFL) photography (Canon), Humphrey Field Analyzer (HFA) with Swedish Interactive Threshold Algorithm (SITA)-Standard 24-2 VF testing (Carl Zeiss Meditec), spectral-domain optical coherence tomography (SD-OCT, Cirrus HD; Carl Zeiss Meditec), and OCT-A (AngioVue; Optovue, Inc., Fremont, CA).

The inclusion criteria for the present study were an open-angle glaucoma (OAG) in patients aged ≥ 18 years at the initial presentation, a BCVA of 20/30 or better, a refractive error of between − 6 to + 3 diopters, and a cylinder correction within + 3 diopters. The enrolled patients were additionally required to have had a minimum of two years follow-up involving at least five serial VF tests, and four good-quality SD-OCT and OCT-A examinations obtained at the same visit during this period. OAG was defined as the presence of an open angle on gonioscopy and glaucomatous ONH damage compatible with a glaucomatous VF defect^13^, regardless of the IOP level. A glaucomatous VF defect was defined in accordance with Anderson’s criteria, as confirmed by at least two initial VF examinations with good reliability (false-positive errors < 15%, false-negative errors < 15%, and fixation loss < 20%)^13^. To account for the learning effect in VF tests, a second VF test was conducted within one week if the first VF test result indicated glaucomatous damage^13^, which was considered to be the baseline test among the serial VF tests. Patients with a VF mean deviation (VF MD) of ≥ − 10 dB were considered to have mild to moderate stage glaucoma^14^ and were included in the study cohort.

Patients were excluded from the study population if they had a history of intraocular surgery except simple cataract surgery, a history of trauma, any retinal vascular/degenerative disease, and severe myopic ONH/macular changes that may induce inaccurate evaluations of ONH/VF/OCT/OCT-A. In addition, eyes/patients with media opacities of more than C2, N2, or P2 during follow-up, as defined by the lens opacities classification system III^15^, which may influence the results of serial VF testing, or displaying systemic or neurologic disease that could influence the ONH/VF evaluations, were also excluded. The affected eye was selected in patients with unilateral disease, while the eye with a poorer VF MD was selected in the cases with bilateral disease.

### Definitions of CVF damage and VF progression

The mild to moderate stage OAG eyes in our current cohort were further selected by the presence of CVF damage at baseline. CVF damage was defined as clusters of three points within the central 10 $^\circ$ with a *P*-value < 0.05, or of up to two points with a *P* < 0.01 on the pattern deviation plot, regardless of the extension from a 10 $^\circ$ to 24 $^\circ$ VF area^16,17^. VF progression was determined by either event- or trend-based analysis. In the event-based analysis, VF progression was defined as a progressive VF change (“likely progression”) at three or more points at the same locations in three consecutive tests^18^, detected using Humphrey field analyser-guided progression analysis software (GPA; Carl Zeiss Meditec). In the trend-based analysis, a significant (*P* < 0.05) negative slope for the VF MD (expressed in dB/yr) was considered to indicate VF progression, since the VF MD is a sensitive index for serial VF analysis, with a relatively short duration of follow-up and number of VF tests required to predict future VF progression in eyes without media opacity^19^.

### ONH/Macular SD-OCT and OCT-A assessments

The study patients underwent optic disc cube and macular scans using a Cirrus SD-OCT (Carl Zeiss Meditec; version 10.0). The average circumpapillary retinal nerve fibre layer thickness (cpRNFLT) was measured in a circle of 3.46 mm in diameter, centred on the ONH. The average macular ganglion cell layer-inner plexiform layer thickness (mGCIPLT) was measured within the circular region having inner horizontal and vertical diameters of 1.2 and 1 mm, respectively, and outer horizontal and vertical diameters of 4.8 and 4 mm, respectively, centred on the fovea. Only SD-OCT scans without segmentation error or motion artifacts with good central fixation and signal strength (SS) ≥ 7 were included in the analysis.

The AngioVue OCT-A imaging system (Optovue Inc.) (software version 2018.1.0.43) was used to assess the microvasculature of the ONH and macular region. The vessel density (VD) was automatically measured using AngioVue software by calculating the percentage of the area occupied by the small vessels. The circumpapillary VD (cpVD) was calculated within the 1000-μm–wide elliptical annulus surrounding the optic disc using ONH imaging of a 4.5 × 4.5 mm^2^ region. This area was centred on the optic disc within the radial peripapillary capillary slab from the internal limiting membrane (ILM) to the nerve fibre layer after the automated removal of large retinal vessels. Macular scans consisted of 6.0 × 6.0 mm^2^ region centred on the fovea. Through the automated segmentation algorithm, both the superficial (from the ILM to the posterior boundary of the inner plexiform layer [IPL]) and deep (from the posterior margin of the IPL to the posterior boundary of the outer plexiform layer [OPL]) layers were analysed. When measuring the mVD in the deep layer, projection artifact removal software was utilized in order to minimize the projection artifact from the retinal vessels of overlying superficial layer. The projection artifact removal algorithm removes projection artifacts based on the normalized voxel-based OCT-A intensity^20^, which is defined as the OCT-A signal intensity per voxel basis divided by OCT intensity. If the normalized OCT-A intensity of a voxel is greater than the normalized OCT-A intensity anterior to the voxel of interest along the axial plane, the voxel is considered to be a real signal and its original OCT-A intensity is maintained; otherwise, the voxel is considered to be a projection artifact and its OCT-A intensity is suppressed to the level of background noise. In each layer on the macular OCT-A images, the mVD was separately analysed from the parafoveal and perifoveal sectors. The parafoveal mVD was measured within an annular region with an inner diameter of 1.0 mm and an outer diameter of 3.0 mm. The perifoveal mVD was measured within an annular region with an inner diameter of 3.0 mm and outer diameter of 6.0 mm. Only good–quality images with an SS of ≥ 7, and without media opacities, motion artifacts, localized weak signal intensities, fixation errors, or segmentation errors, were included in the study.

### Statistical analysis

Demographics and clinical characteristics were compared between the eyes with and without VF progression using an independent *t*-test for normally distributed data or the Mann–Whitney U test for continuous variables, based on normality testing using the Kolmogorov–Smirnov test. For categorical variables, the Fisher's exact test or chi-squared test was used as appropriate. The rates of change in the mVD and structural parameters over time were analysed and compared between the VF progressors and non-progressors using a linear mixed effects model. This model was fitted using fixed effects with age, number of tests, scan quality, follow-up duration, CCT, axial length, baseline IOP, mean follow-up IOP, and baseline VF MD, accepting random intercepts and coefficients at the individual level when analysing the effects of time.

The clinical factors associated with VF progression based on either event- or trend-based analysis were evaluated using Cox regression analyses and multivariable models with a backward elimination approach were built using variables showing *P* < 0.05 in the univariable analysis. The backward elimination approach was performed by checking all variables in the univariable model and selected those, which had *P* < 0.05. The next step was that these significant variables (*P* < 0.05) were considered in a multivariable model with backward selection. Variables with nonsignificant *P*-values at the level of 0.05 were removed from the model. The reduced model should then include the best explanatory variables. Linear regression analyses were conducted to determine the clinical factors associated with trend analysis based VF MD reduction rate. Variables with a *P* < 0.05 in the univariable analysis were entered into the multivariable model via a stepwise elimination approach. By using the stepwise elimination method, variables were added one by one, until no further variables could be added to improve the statistical significance of the model fit. Survival outcomes (time to confirmed VF progression) as a function of the mVD and mGCIPLT reduction rates were assessed using Kaplan–Meier survival analyses. Log-rank tests were applied to compare the groups having the upper and lower 50th percentiles of reduction rates. The Pearson correlation analysis was performed to assess the relationship between the rates of change in superficial and deep layer mVD parameters and the reduction rates of VF MD. Statistical analyses were performed using SPSS software, version 21.0 (IBM Corp, Armonk, NY). A *P* value of 0.05 or less was considered to indicate statistical significance.

## Results

Based on the application of our inclusion criteria, a total of 182 eyes from 182 mild to moderate stage glaucoma patients were included in this retrospective longitudinal study, 48 of which (26.4%) showed VF progression during a mean follow-up of 3.5 years. In the VF progressor group, the number of eyes that showed VF progression according to event-based analysis, trend-based analysis, and both methods were 43 (89.6%), 38 (79.2%), and 33 (68.8%), respectively. Average numbers of VF, SD-OCT, and OCT-A tests for each eye were 6.5, 5.2, and 5.2, respectively.

Table 1 summarizes the demographics and the clinical characteristics of the entire OAG eye cohort and in the subgroups with and without VF progression. There were no statistical differences in age, gender, axial length, CCT, follow-up duration, baseline VF MD or visual field index (VFI) between the two groups. Despite that absence of significant differences in the baseline VF MD or VFI parameters, the VF progressor group showed a significantly lower cpVD (*P* = 0.003), lower superficial layer mVDs of the parafoveal (*P* = 0.006) and perifoveal (*P* = 0.049) sectors, and both a thinner cpRNFLT (*P* = 0.037) and mGCIPLT (*P* = 0.012) at the final visit. The deep layer mVD parameters did not show any significant differences between the VF progressor and non-progressor groups at either the baseline or final visits.

**Table 1:** Clinical characteristics of open-angle glaucoma eyes with and without visual field progression.

	Entire Cohort (n = 182)	VF Progressor (n = 48)	VF Non-progressor (n = 134)	*P* value*
Age, yr	57.68 ± 11.76	60.10 ± 12.97	56.81 ± 11.22	0.096
Gender, M / F	96 / 86	22 / 26	74 / 60	0.265
Axial length, mm	24.86 ± 1.50	24.52 ± 1.58	24.99 ± 1.46	0.096
Central corneal thickness, µm	533.49 ± 39.75	535.33 ± 41.14	532.83 ± 39.40	0.726
Follow-up duration, yr	3.50 ± 0.75	3.57 ± 0.75	3.48 ± 0.74	0.495
Quality of OCT-A scans	7.45 ± 0.67	7.31 ± 0.56	7.49 ± 0.70	0.127
Quality of OCT scans	7.53 ± 0.64	7.48 ± 0.62	7.54 ± 0.65	0.561
Hypertension, n (%)	37 (20.3%)	13 (27.1%)	24 (17.9%)	0.201
Diabetes mellitus, n (%)	16 (8.8%)	5 (10.4%)	11 (8.2%)	0.657
Anti-glaucoma medication, n	1.61 ± 0.93	1.75 ± 0.96	1.53 ± 0.91	0.187
*IOP measurements*				
Baseline IOP	15.73 ± 3.91	16.56 ± 5.03	15.43 ± 3.40	0.154
Follow-up mean IOP	13.55 ± 1.76	13.75 ± 2.18	13.47 ± 1.59	0.433
Follow-up peak IOP	16.34 ± 3.83	17.58 ± 5.54	15.90 ± 2.89	**0.049**
*VF measurements*				
Baseline MD, dB	− 4.91 ± 2.68	− 5.17 ± 2.69	− 4.81 ± 2.68	0.423
Final MD, dB	− 7.42 ± 4.12	− 10.66 ± 5.121	− 6.25 ± 2.95	** < 0.001**
Baseline VFI, %	84.25 ± 8.24	84.08 ± 8.63	84.31 ± 8.12	0.869
Final VFI, %	76.31 ± 13.65	66.48 ± 17.19	79.83 ± 10.08	** < 0.001**
*cpVD, %*				
Baseline cpVD, %	43.14 ± 5.42	42.06 ± 5.62	43.53 ± 5.31	0.111
Final cpVD, %	40.85 ± 6.02	38.66 ± 6.52	41.64 ± 5.66	**0.003**
*Superficial parafoveal mVD, %*				
Baseline superficial parafoveal mVD, %	44.30 ± 5.98	44.68 ± 5.77	44.16 ± 6.07	0.607
Final superficial parafoveal mVD, %	42.06 ± 5.53	40.19 ± 5.38	42.74 ± 5.45	**0.006**
*Superficial perifoveal mVD, %*				
Baseline superficial perifoveal mVD, %	40.13 ± 4.40	40.70 ± 4.64	39.92 ± 4.31	0.294
Final superficial perifoveal mVD, %	38.11 ± 4.05	37.12 ± 4.28	38.46 ± 3.93	**0.049**
*Deep parafoveal mVD, %*				
Baseline deep parafoveal mVD, %	51.29 ± 5.99	51.78 ± 5.77	51.11 ± 6.09	0.509
Final deep parafoveal mVD, %	50.03 ± 6.09	49.76 ± 6.04	50.11 ± 6.13	0.748
*Deep perifoveal mVD, %*				
Baseline deep perifoveal mVD, %	46.59 ± 6.51	47.22 ± 5.84	46.36 ± 6.74	0.431
Final deep perifoveal mVD, %	44.64 ± 7.24	44.29 ± 6.78	44.76 ± 7.40	0.720
*cpRNFLT, µm*				
Baseline cpRNFLT, µm	71.36 ± 8.80	70.44 ± 9.11	71.73 ± 8.69	0.386
Final cpRNFLT, µm	70.54 ± 8.90	68.77 ± 9.55	71.21 ± 8.59	**0.037**
*mGCIPLT, µm*				
Baseline mGCIPLT, µm	66.24 ± 7.07	65.44 ± 8.43	66.54 ± 6.52	0.417
Final mGCIPLT, µm	64.04 ± 7.14	61.56 ± 8.09	64.93 ± 6.58	**0.012**

*An independent *t*-test was used for normally distributed data and Mann–Whitney U test for categorical variables. Values are expressed as a mean ± standard deviation. Values with statistical significance are presented in bold.

*OCT-A* optical coherence tomography angiography, *OCT* optical coherence tomography, *IOP* intraocular pressure, *VF* visual field, *MD* mean deviation, *VFI* visual field index, *cpVD* circumpapillary vessel density, *mVD* macular vessel density, *cpRNFLT* circumpapillary retinal nerve fibre layer thickness, *mGCIPLT* macular ganglion cell-inner plexiform layer thickness.

The rates of change in the mVD and thickness parameters during follow-up period are presented in Table 2. In the entire cohort, the cpVD, superficial and deep layer mVDs of both parafoveal and perifoveal sectors, cpRNFLT, and mGCIPLT showed significant decreases over time (*P* < 0.05). In comparative analyses (i.e., VF progressors vs. non-progressors), the rates of change in both superficial and deep layer mVD parameters were significantly faster in the eyes with VF progression (*P* < 0.05) at both the parafoveal and perifoveal sectors. The VF progressors also showed significantly faster rates of loss in the cpRNFLT (*P* = 0.013), mGCIPLT (*P* < 0.001), and VF MD (*P* < 0.001) compared to the non-progressors.

**Table 2:** Rates of change in the macular vessel density, circumapapillary retinal nerve fibre layer thickness, macular ganglion cell inner plexiform layer thickness, and visual field mean deviation between eyes with and without visual field progression determined using linear mixed effects models.

Variables	Entire Cohort (n = 182)		VF Progressors (n = 48)	VF Non-progressors (n = 134)	
	Slope (95% CI)	*p*-value	Slope (95% CI)	Slope (95% CI)	*p*-value*
cpVD change rate, %/yr	− 0.465 (− 0.561–− 0.369)	** < 0.001**	− 0.770 (− 0.986–− 0.554)	− 0.357 (− 0.461–− 0.254)	** < 0.001**
Superficial parafoveal mVD change rate, %/yr	− 0.428 (− 0.574–− 0.282)	** < 0.001**	− 0.827 (− 1.113–− 0.541)	− 0.289 (− 0.456–− 0.122)	** < 0.001**
Superficial perifoveal mVD change rate, %/yr	− 0.394 (− 0.495–− 0.293)	** < 0.001**	− 0.653 (− 0.870–− 0.436)	− 0.302 (− 0.415–− 0.189)	** < 0.001**
Deep parafoveal mVD change rate, %/yr	− 0.300 (− 0.481–− 0.118)	**0.001**	− 0.527 (− 0.929–− 0.125)	− 0.228 (− 0.431–− 0.025)	**0.002**
Deep perifoveal mVD change rate, %/yr	− 0.449 (− 0.661–− 0.238)	** < 0.001**	− 0.708 (− 1.074–− 0.342)	− 0.358 (− 0.609–− 0.106)	**0.001**
cpRNFLT change rate, µm/yr	− 0.138 (− 0.250–− 0.026)	**0.016**	− 0.448 (− 0.728–− 0.168)	− 0.007 (− 0.126–0.111)	**0.002**
mGCIPLT change rate, µm/yr	− 0.401 (− 0.492–− 0.309)	** < 0.001**	− 0.803 (− 1.044–− 0.562)	− 0.279 (− 0.373–− 0.185)	** < 0.001**
VF MD change rate, dB/yr	− 0.497 (− 0.591–− 0.404)	** < 0.001**	− 1.097 (− 1.311–− 0.883)	− 0.288 (− 0.354–− 0.222)	** < 0.001**

The longitudinal rates of change were calculated with a linear mixed model, using the fixed effects of age, axial length, CCT, scan quality, follow-up period, number of tests, baseline IOP, and baseline VF MD. Values with statistical significance are presented in bold.

*The rates of change in each clinical parameter were compared between the VF progressors and non-progressors using linear mixed effect models.

*VF* visual field, *cpVD* circumpapillary vessel density, *mVD* macular vessel density, *cpRNFLT* circumpapillary retinal nerve fibre layer thickness, *mGCIPLT* macular ganglion cell-inner plexiform layer thickness.

The clinical factors related to the VF progression were evaluated using Cox regression analyses (Table 3). Two separate sets of multivariable analyses were conducted to avoid multicollinearity between the rates of superficial parafoveal and perifoveal mVDs as there were strong correlations between the two parameters due to the close proximity of anatomical locations (*r* = 0.772, *P* < 0.001; Pearson correlation analysis)^21,22^. In multivariable model 1, including the rate of change in the superficial layer parafoveal mVD, more rapid cpVD loss (hazard ratio [HR] 0.563, *P* = 0.009) and greater reduction rates in the superficial layer parafoveal mVD (HR 0.651, *P* = 0.006) and cpRNFLT (HR 0.675, *P* = 0.032) showed significant associations with VF progression. In multivariable model 2, including the rate of change in the superficial layer perifoveal mVD, greater reduction rates in the superficial layer perifoveal mVD (HR 0.579, *P* = 0.018), cpRNFLT (HR 0.688, *P* = 0.033), and mGCIPLT (HR 0.595, *P* = 0.015) were significantly associated with VF progression.

**Table 3:** Univariable and multivariable Cox regression analyses to identify clinical factors associated with visual field progression.

Variables	Entire cohort (n = 182)								
	Univariable analysis			Multivariable analysis					
				Model 1			Model 2		
	HR	95% CI	*p*-value	HR	95% CI	*p*-value	HR	95% CI	*p*-value
Age, years	1.022	0.995–1.049	0.108						
Gender	1.418	0.803–2.502	0.229						
Axial length, mm	0.830	0.660–1.044	0.111						
Central corneal thickness, µm	1.001	0.993–1.009	0.856						
Baseline IOP, mmHg	1.054	0.990–1.122	0.098						
Follow-up mean IOP, mmHg	1.082	0.920–1.272	0.343						
Follow-up peak IOP, mmHg	1.089	1.027–1.154	**0.004**	Dropped			Dropped		
Baseline VF MD	0.976	0.879–1.083	0.647						
Baseline cpVD, %	0.962	0.915–1.012	0.962						
Baseline superficial parafoveal VD, %	1.009	0.963–1.058	0.707						
Baseline superficial perifoveal VD, %	1.033	0.967–1.104	0.333						
Baseline deep parafoveal VD, %	1.022	0.974–1.072	0.374						
Baseline deep perifoveal VD, %	1.022	0.979–1.068	0.320						
Baseline cpRNFLT, µm	0.993	0.961–1.025	0.648						
Baseline mGCIPLT, µm	0.980	0.940–1.021	0.331						
cpVD change rate, %/yr	0.482	0.323–0.719	** < 0.001**	0.563	0.366–0.866	**0.009**	Dropped		
Superficial parafoveal mVD change rate, %/yr	0.613	0.454–0.828	**0.001**	0.651	0.479–0.885	**0.006**			
Superficial perifoveal mVD change rate, %/yr	0.491	0.317–0.762	**0.001**				0.579	0.367–0.912	**0.018**
Deep parafoveal mVD change rate, %/yr	0.980	0.800–1.201	0.846						
Deep perifoveal mVD change rate, %/yr	0.937	0.778–1.127	0.490						
cpRNFLT change rate, µm/yr	0.535	0.381–0.753	** < 0.001**	0.675	0.472–0.966	**0.032**	0.688	.0488–0.970	**0.033**
mGCIPLT change rate, µm/yr	0.492	0.341–0.708	** < 0.001**	Dropped			0.595	0.391–0.903	**0.015**

Values with statistical significance are presented in bold.

Model 1: Follow-up peak IOP, cpVD rate, superficial parafoveal VD rate, cpRNFLT rate, mGCIPLT rate.

Model 2: Follow-up peak IOP, cpVD rate, superficial perifoveal VD rate, cpRNFLT rate, mGCIPLT rate.

*IOP* intraocular pressure, *VF* visual field, *MD* mean deviation, *mVD* macular vessel density, *cpVD* circumpapillary vessel density, *cpRNFLT* circumpapillary retinal nerve fibre layer thickness, *mGCIPLT* macular ganglion cell-inner plexiform layer thickness.

Linear regression analyses were performed to assess the clinical factors associated with a faster VF MD reduction rate (Table 4). Multivariable model 1, including the rate of change in the superficial layer parafoveal mVD, revealed that a higher follow-up peak IOP (β = − 0.045, *P* < 0.001), lower baseline cpVD (β = 0.021, *P* = 0.010), and greater reduction rates of cpVD (β = 0.243, *P* = 0.001), superficial layer parafoveal mVD (β = 0.090, *P* = 0.040), and mGCIPLT (β = 0.193, *P* = 0.010), were significantly associated with more rapid VF progression. In the multivariable model 2 including rate of change in superficial layer perifoveal mVD, greater reduction rate of superficial perifoveal mVD (β = 0.129, *P* = 0.048) as well as higher follow-up peak IOP (β = − 0.043, *P* < 0.001), lower baseline cpVD (β = 0.023, *P* = 0.004), and greater reduction rates of cpVD (β = 0.226, *P* = 0.001) and mGCIPLT (β = 0.201, *P* = 0.007), showed significant correlations with the faster rate of VF progression. None of the deep layer mVD parameters, including the baseline value or the rate of change, showed any significant association with VF progression or the rate of VF progression.

**Table 4:** Univariable and multivariable linear regression analysis to identify the clinical factors associated with a visual field mean deviation reduction rate.

Variables	Entire cohort (n = 182)								
	Univariable analysis			Multivariable analysis					
				Model 1			Model 2		
	β	95% CI	*p*-value	β	95% CI	*p*-value	β	95% CI	*p*-value
Age, years	− 0.003	− 0.011–0.004	0.368						
Gender	0.048	− 0.132–0.227	0.602						
Axial length, mm	0.015	− 0.055–0.085	0.676						
Central corneal thickness, µm	0.001	− 0.002–0.003	0.575						
Baseline IOP, mmHg	− 0.014	− 0.037–0.009	0.217						
Follow-up mean IOP, mmHg	− 0.089	− 0.138–− 0.039	** < 0.001**	Dropped			Dropped		
Follow-up peak IOP, mmHg	− 0.061	− 0.083–− 0.040	** < 0.001**	− 0.045	− 0.066–− 0.023	** < 0.001**	− 0.043	− 0.064–− 0.021	** < 0.001**
Baseline VF MD	0.008	− 0.026–0.041	0.658						
Baseline cpVD, %	0.026	0.009–0.042	**0.002**	0.021	0.005–0.036	**0.010**	0.023	0.007–0.038	**0.004**
Baseline superficial parafoveal VD, %	0.012	− 0.003–0.027	0.128						
Baseline superficial perifoveal VD, %	0.013	− 0.007–0.034	0.196						
Baseline deep parafoveal VD, %	0.004	− 0.011–0.019	0.594						
Baseline deep perifoveal VD, %	0.006	− 0.008–0.020	0.422						
Baseline cpRNFLT, µm	0.003	− 0.007–0.014	0.560						
Baseline mGCIPLT, µm	0.016	0.003–0.029	**0.013**	Dropped			Dropped		
cpVD change rate, %/yr	0.288	0.154–0.421	** < 0.001**	0.243	0.103–0.383	**0.001**	0.226	0.090–0.363	**0.001**
Superficial parafoveal mVD change rate, %/yr	0.131	0.035–0.227	**0.008**	0.090	0.004–0.176	**0.040**			
Superficial perifoveal mVD change rate, %/yr	0.216	0.074–0.358	**0.003**				0.129	0.001–0.257	**0.048**
Deep parafoveal mVD change rate, %/yr	0.039	− 0.022–0.101	0.210						
Deep perifoveal mVD change rate, %/yr	0.042	− 0.014–0.099	0.139						
cpRNFLT change rate, µm/yr	0.213	0.070–0.355	**0.004**	Dropped			Dropped		
mGCIPLT change rate, µm/yr	0.319	0.175–0.463	** < 0.001**	0.193	0.048–0.338	**0.010**	0.201	0.057–0.345	**0.007**

Values with statistical significance are presented in bold.

Model 1: Follow-up mean IOP, follow-up peak IOP, baseline cpVD, baseline mGCIPLT, cpVD rate, superficial parafoveal VD rate, cpRNFLT rate, mGCIPLT rate.

Model 2: Follow-up mean IOP, follow-up peak IOP, baseline cpVD, baseline mGCIPLT, cpVD rate, superficial perifoveal VD rate, cpRNFLT rate, mGCIPLT rate.

*IOP* intraocular pressure, *VF* visual field, *MD* mean deviation, *mVD* macular vessel density, *cpVD* circumpapillary vessel density, *cpRNFLT* circumpapillary retinal nerve fibre layer thickness, *mGCIPLT* macular ganglion cell-inner plexiform layer thickness.

**Figure 1:**
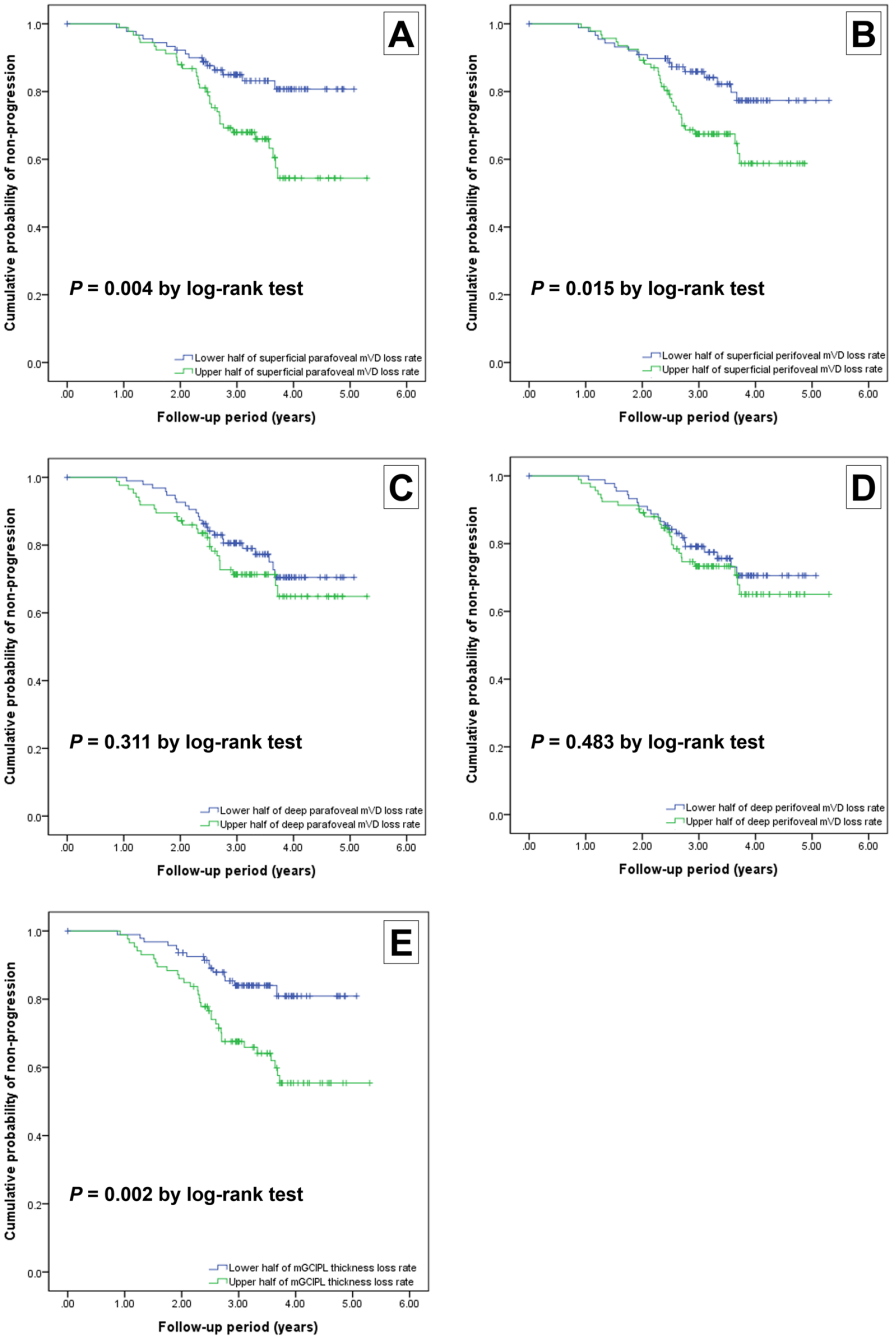
Kaplan–Meier survival curves indicating the effects of macular vessel density (mVD) change rates and thickness parameters (superficial and deep layer parafoveal and perifoveal mVD change rates and macular ganglion cell-inner plexiform layer thickness [mGCIPLT] change rate) on visual field (VF) progression in mild to moderate stage open-angle glaucoma (OAG) eyes with central VF damage. Survival curves of the upper and lower 50th percentiles were compared using log-rank tests. Significant differences were found between eyes in the upper and lower half of the superficial layer parafoveal mVD reduction rate (**A**; *P* = 0.004, log-rank test). Differences in the probability of VF progression were found to be statistically significant between eyes in the upper and lower half of superficial layer perifoveal mVD reduction rate (**B**; *P* = 0.015, log-rank test). Comparison of probability of VF progression based on the deep layer parafoveal (**C**; *P* = 0.311, log-rank test) and perifoveal (**D**; *P* = 0.483, log-rank test) mVD reduction rates did not show significant differences (*P* > 0.05). Differences in the probability of VF progression were statistically significant between the upper and lower half of the mGCIPLT reduction rate (**E**; *P* = 0.002, log-rank test).

Figure 1 presents Kaplan–Meier survival curves for the effects of changes in the superficial and deep layer mVD and mGCIPLT parameters on subsequent VF progression. Survival curves of the groups stratified into an upper and lower 50th percentiles for each parameter were compared using log-rank tests. The probability of VF progression was significantly higher in eyes in the upper halves of the superficial layer parafoveal (*P* = 0.004) and perifoveal (*P* = 0.015) mVD reduction rates, and mGCIPLT decrease rate (*P* = 0.002). No significant differences were found between eyes in the upper and lower halves of the deep layer parafoveal (*P* = 0.311) and perifoveal (*P* = 0.483) mVD loss rate.

**Figure 2:**
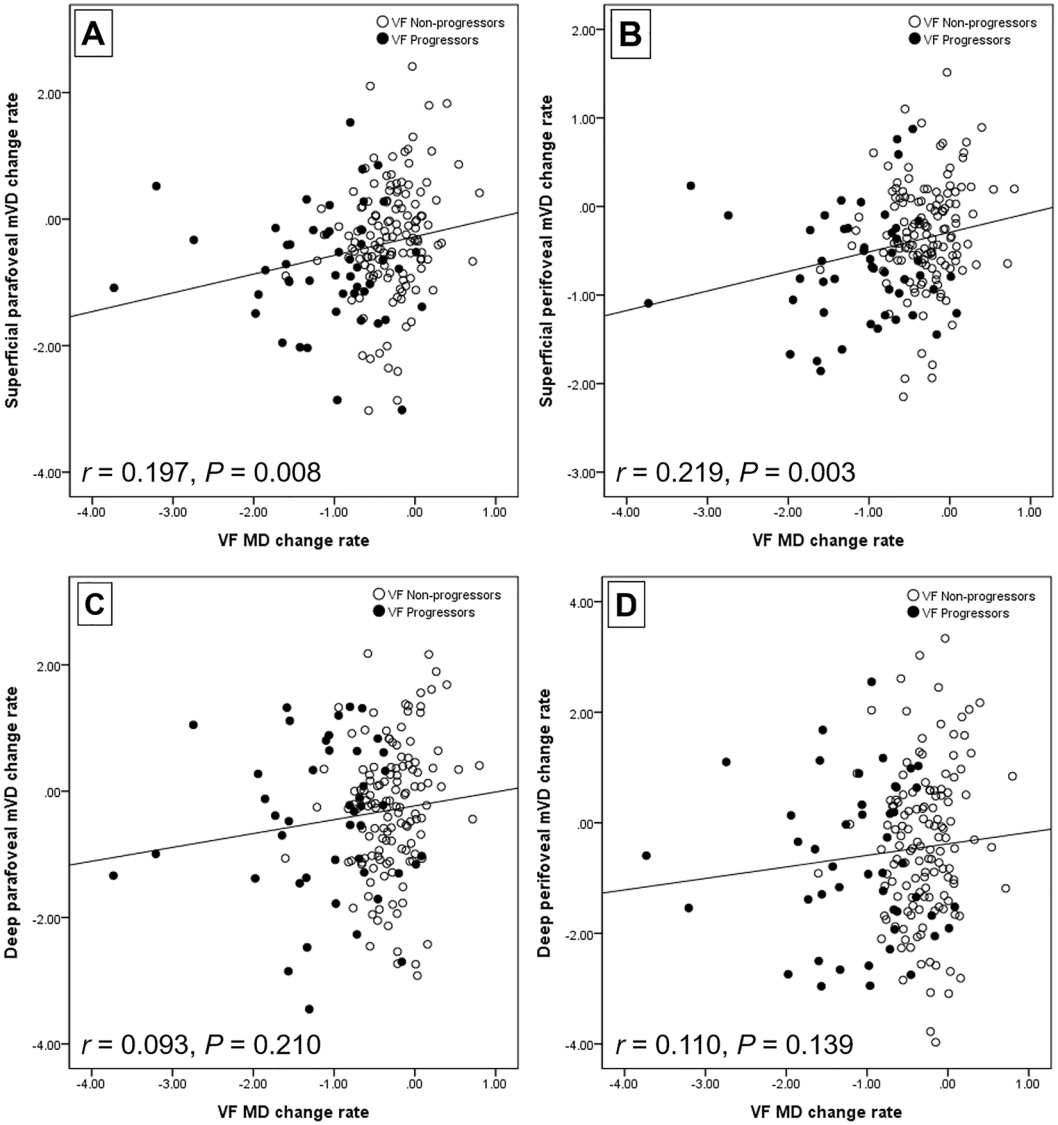
Scatter plots demonstrating the relationship between the rates of change in superficial/deep mVD parameters and VF MD reduction rates. The superficial parafoveal (**A**) and perifoveal (**B**) mVD loss rates showed significant correlations with the reduction rates of VF MD (*P* = 0.008 and *P* = 0.003, respectively; Pearson correlation analysis), while the rates of change in deep layer mVD at parafoveal (**C**) and perifoveal (**D**) area were not significantly related to the reduction rates of VF MD (*P* = 0.210 and *P* = 0.139, respectively; Pearson correlation analysis).

Figure 2 demonstrates the relationship between the rates of change in superficial/deep mVD parameters and VF MD. The Pearson correlation analyses showed significant associations between the rates of superficial layer parafoveal and perifoveal mVD loss and the reduction rate of VF MD (*P* = 0.008 and *P* = 0.003, respectively), while the rates of deep layer mVD loss in parafoveal and perifoveal area were not related to the VF MD loss rate (*P* = 0.210 and *P* = 0.139, respectively).

**Figure 3:**
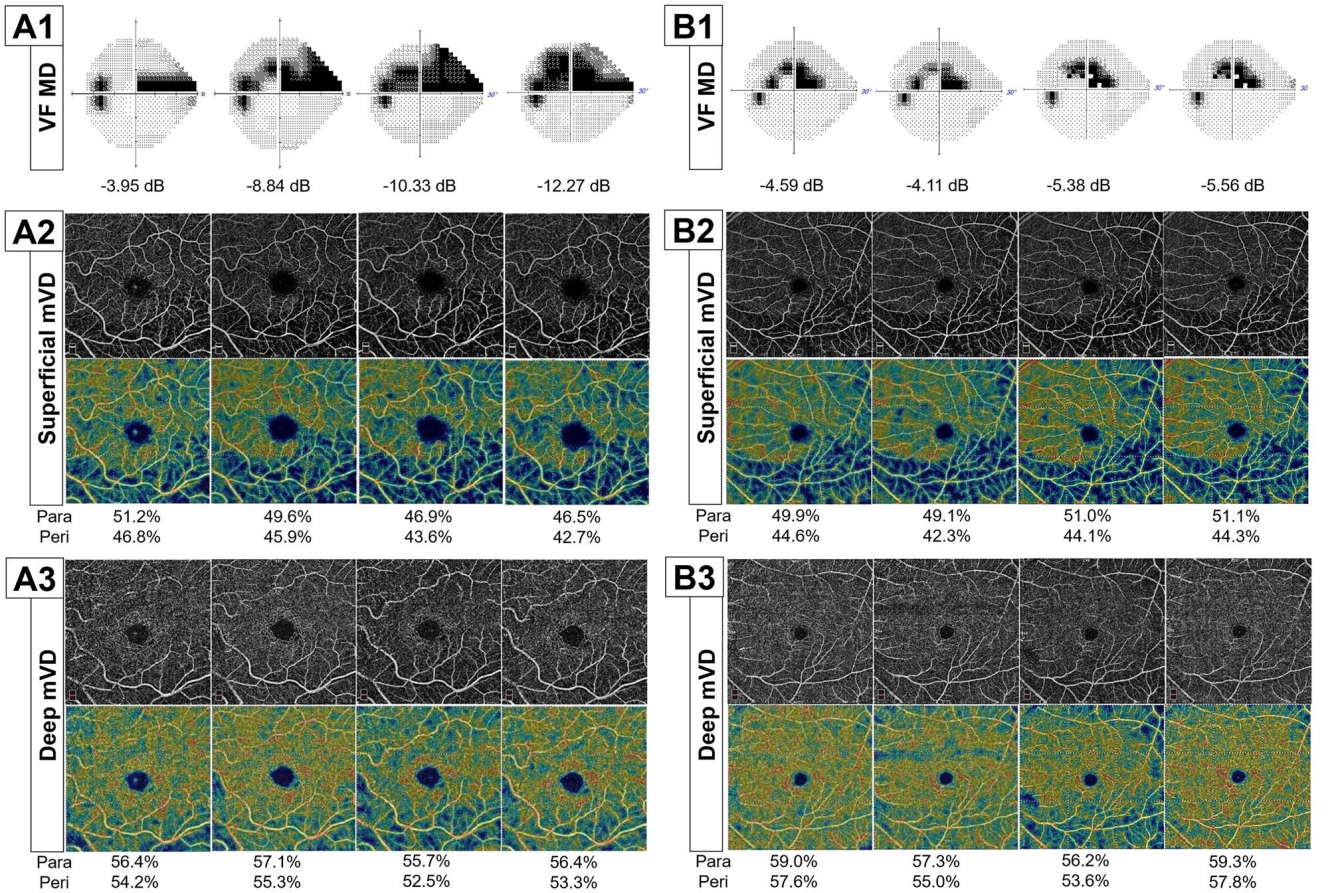
Representative cases (**A, B**) of mild to moderate stage open-angle glaucoma (OAG) eyes with and without visual field (VF) progression. (**A**) A 63-year-old female OAG patient showed central VF (CVF) defects at baseline and progressive VF loss in the superior hemifield (**A1**). En face and color-coded thickness macular maps of the superficial layer derived from optical coherence tomography angiography (OCT-A) indicated an obvious macular vessel density (mVD) attenuation at the inferior hemiretina throughout the follow-up period (**A2**). The En face and color-coded thickness macular maps of the deep layer derived from the OCT-A scans indicated a stable mVD throughout the follow-up (**A3**). (**B**) A 57-year-old female OAG patient showed CVF defects at baseline and a stable VF during follow-up (**B1**). The mVD remained unchanged in this patient throughout the follow-up period, as indicated by En face and color-coded macular thickness maps of the superficial layer derived from the OCT-A images (**B2**). Moreover, there was no evidence of an mVD reduction throughout the follow-up on the En face and color-coded thickness macular maps of the deep layer derived from the OCT-A scan (**B3**).

Figure 3 shows representative cases with and without VF progression during follow-up in OAG eyes with CVF damage, illustrating a stronger association between the reduction rates of superficial layer parafoveal and perifoveal mVD parameters and concurrent VF progression than between deep layer parafoveal and perifoveal mVD reduction and subsequent VF progression.

## Discussion

Our present study findings have demonstrated the longitudinal changes in parafoveal and perifoveal mVD parameters at different layers, and their association with concomitant progressive VF loss in mild to moderate stage OAG eyes with baseline CVF damage. Our current data have indicated that the development and the rate of VF progression are significantly associated with a more rapid decrease in superficial but not deep layer mVD parameters. These findings thus suggest that there is a potential use for superficial mVD parameters in predicting glaucomatous VF progression, and highlight the importance of monitoring superficial rather than deep layer mVD changes in mild to moderate stage OAG eyes with CVF damage.

It must be noted that there are previous reports of the usefulness of the deep layer mVD for detecting glaucoma progression^6,7^. Jeon et al.^6^ reported that the deep layer mVD was significantly correlated with central VF parameters, such as the VF MD of SITA 10-2 testing. According to their study findings using multivariable regression analysis, a significant risk factor affecting central visual function was deep rather than superficial layer mVD loss. This discrepancy with our current results can be explained by differences in the study design, in the imaging devices used to measure mVD, and/or the study patients enrolled. Our present patient series consisted of mild to moderate stage OAG eyes with CVF loss at baseline that were analysed to assess a possible association between mVD reduction at superficial and deep layers and concomitant VF progression. Moreover, prior studies^6,7^ have used a different OCT-A instrument (DRI OCT Triton; Topcon) and no projection artifacts removal system was applied when measuring the deep layer mVD in those investigations. Recent studies^5,9,12^ that did use a projection artifacts removal system have reported preferential glaucomatous damage in the SVP rather than DVP, which is consistent with our present findings.

In our present analyses, the reduction rates of mVD parameters in both the superficial and deep layers were significantly greater in the VF progressors than in the non-progressors, but only the superficial layer mVD changes were found to be clinically relevant to this VF progression or to the rate of VF deterioration. Glaucomatous damage may affect both superficial and deep layer mVD^8,9^, as shown by significantly faster rates of superficial and deep layer mVD loss in the VF progressors than in the non-progressors. Nonetheless, our findings showed significant associations between the rates of superficial, not deep, layer mVD loss and VF progression and the rate of VF decay. Our findings are consistent with the previous studies showing that superficial layer mVD reflects the impact of glaucomatous damage more sensitively than the deep layer mVD^5,8,9,12^. In these prior reports, the superficial layer mVD also showed better diagnostic capability in relation to glaucoma than the deep layer mVD^5,12^, and only the superficial layer mVD was found to be significantly reduced in early OAG patients with CVF damage^9^. These findings suggest that superficial layer mVD loss occurs preferentially in the early glaucoma stages and that the superficial layer mVD may be more informative than the deep layer mVD in detecting glaucomatous changes. The preferential damage to superficial layer mVD parameters in early-stage glaucoma can be explained by the anatomy of the vascular structure. The superficial layer mVD depicts the VD of the SVP, which supplies the RNFL and RGCs, while the deep layer mVD relates to the perfusion of the DVP supplying the horizontal cells in the outer nuclear layer^23^. Considering that the RNFL and RGCs are the primary sites for glaucomatous structural damage^24^, glaucomatous change may be more closely related to changes in the SVP than in the DVP, and hence, superficial layer mVD parameters can be more predictive of glaucomatous damage and progression.

Our current study findings are in line with previous results that revealed a significant correlation between superficial layer mVD reduction and glaucomatous damage^7,8,25^. Hou et al.^24^ reported that the superficial layer mVD decreased more rapidly in primary open-angle glaucoma eyes compared to preperimetric glaucoma or healthy eyes. This implies that a faster decrease in the superficial layer mVD is associated with more severe glaucomatous damage. Kamalipour et al.^8^ recently showed that a lower superficial layer mVD was associated with the development and faster rate of past VF loss. The assessment of glaucomatous VF progression in that study, however, was limited to past changes that occurred prior to the OCT-A acquisition, and the OCT-A-derived parameters were assessed within six months before the study endpoint in a cross-sectional manner. While our present study also confirmed that glaucomatous eyes with a rapid loss of superficial layer mVD were prone to VF progression, our current data are of greater clinical relevance as they demonstrate a significant relationship between serial changes in superficial layer mVD loss and concurrent VF progression, and that the reduction rates in the superficial layer mVD parameters are linearly associated with the VF progression rate, as determined by VF MD decay.

Along with the superficial layer mVD, the reduction rate of the cpVD also showed a significant correlation with the VF progression in our current analyses. It is well known that the cpVD is lower in glaucomatous eyes than in healthy eyes^26,27^. Moreover, the cpVD was found previously to be significantly associated with VF mean sensitivity of the corresponding area in glaucomatous eyes, indicating a strong vascular-functional relationship between them^28^. A recent study by Shin et al^11^. further reported a significant association between the rate of cpVD reduction and VF progression, regardless of the glaucoma severity. Since the macula contains more than 50% of the RGCs and RNFL that travel to the temporal side of the circumpapillary region^29^, a fast rate of cpVD loss would be expected to be associated with VF progression as well as faster rate of VF deterioration noted in our present series of OAG eye with CVF scotoma. This suggests that monitoring cpVD loss, in addition to the superficial layer mVD, may be also valuable in predicting the VF progression in mild to moderate stage OAG eyes with CVF damage.

Our present study findings have revealed that the reduction rate of the mGCIPLT is related to the likelihood and rate of VF progression. Earlier studies reported that glaucoma results in the thinning of both the circumpapillary retinal nerve fibre (cpRNFL) and macular RGC layers, but that the RGC layer was notably thinner^30–32^, especially in eyes with parafoveal VF loss^33^. Another study revealed that VF progression in OAG patients with CVF damage is related to a thinner macular ganglion cell-inner plexiform layer (mGCIPL), but not cpRNFL, at baseline, which may be explained by the topographic disparity between the cpRNFL and CVF sensitivity^10^. Our present study cohort consisted of OAG eyes with CVF damage, and it is not surprising to note that the VF progression of CVF damage may be therefore reflected by the corresponding glaucomatous macular damage (i.e., mGCIPL). In the current study, the reduction rates of cpVD and cpRNFLT were related to the likelihood of subsequent VF progression in our OAG patients with CVF damage according to Cox regression analyses. These correlations may be explained by the possibility that additional VF damage could have occurred outside the CVF region in some eyes with more extensive CVF damage during follow-up, even though our study included only OAG eyes with CVF damage at baseline. As glaucoma progresses in the OAG eyes with CVF defects, VF progression may involve the expansion of VF damage outside CVF area as well as deepening of existing CVF deficit, resulting in our finding that the glaucomatous VF progression was significantly associated with progressive loss of cpVD/cpRNFLT and mGCIPLT.

The rate of VF MD reduction in the entire study cohort was − 0.497 dB/yr and VF progressors showed an average rate of VF MD reduction of − 1.097 dB/yr in the current study. While average VF MD reduction rate of the present study is in line with those of previous studies^34,35^, which reported global VF MD change rate to be between − 0.3 and − 0.58 dB/yr, the relatively high rate of VF progression in our progressor group (− 1.10 dB/yr) may be due to some patients (n = 9) with high peak IOP (> 21 mmHg, average peak IOP 27.22 ± 4.89 mmHg) during follow-up despite medical treatment, whose average VF MD change rate was − 2.15 dB/yr. Another explanation for relatively high rate of VF progression among our OAG progressors may be related to the existence of vascular mechanism. The presence of vascular insufficiency in these normal-tension glaucoma (NTG) eyes may also contribute to faster rate of VF progression despite having normal follow-up IOPs.

This study had several limitations of note. First, the vascularity of the deep layer is especially vulnerable to projection artifacts from the superficial layer retinal vessels. Hence, the possibility of residual projection artifacts may exist despite our use of the removal algorithm^5^. Second, glaucoma can eventually affect all layers of the capillary plexus^36–38^, and both superficial and deep layer mVD parameters can be involved as the disease progresses into more advanced stages^9^. Our study population consisted of mild to moderate stage OAG patients (VF MD ≥ − 10 dB) with CVF and different results can be found between moderate and advanced stage glaucoma cases. Further studies on a series comprising a greater range of glaucoma stages may be required to finalize the association between mVD parameters in different layers (superficial vs deep) and VF progression. Third, certain glaucoma medications such as β-blockers are known to affect and reduce the superficial layer mVD^5^. Vasoconstriction induced by β-blockers may therefore have affected our study results and our current findings should be cautiously interpreted. Fourth, the 10-2 VF may be more informative in the monitoring of glaucoma patients with initial CVF damage due to its greater spatial information compared to 24-2 VF^39^. Nonetheless, since OAG patients with initial CVF damage were recruited to our present study cohort and followed for VF progression and its rate, the 24-2 VF modality may offer a wider area of VF testing and may be more suitable for evaluating subsequent VF progression during follow-up. Finally, our study was based on the retrospective design with the patients recruited from a tertiary university hospital, which may have caused a selection bias and our findings may therefore not have full general applicability.

In conclusion, the reduction rates of the superficial layer parafoveal and perifoveal mVDs are higher in VF progressors compared to non-progressors in mild to moderate stage OAG eyes with CVF defects. However, only the rates of change in the superficial layer parafoveal and perifoveal mVDs are significantly associated with a higher likelihood and rate of VF progression. These findings suggest that a rapid decrease in the superficial rather than deep-layer mVD parameters, measured with OCT-A, may be useful in detecting and monitoring glaucomatous VF progression in mild to moderate stage OAG eyes with CVF defects.

## Data Availability

The datasets generated and analysed during the current study are available from the corresponding author on reasonable request.
